# Genomic investigation of duplication, functional conservation, and divergence in the LRR-RLK Family of* Saccharum*

**DOI:** 10.1186/s12864-024-10073-z

**Published:** 2024-02-09

**Authors:** Hongyan Ding, Xiaoxi Feng, Yuan Yuan, Baiyu Wang, Yuhao Wang, Jisen Zhang

**Affiliations:** 1 State Key Laboratory for Conservation and Utilization of Subtropical Agro-Bioresources and Guangxi Key Lab for Sugarcane Biology, Guangxi University, Nanning, 530004 China; 2 Center for Genomics and Biotechnology, Fujian Provincial Key Laboratory of Haixia Applied Plant Systems Biology, Key Laboratory of Sugarcane Biology and Genetic Breeding, National Engineering Research Center for Sugarcane, Fujian Agriculture and Forestry University, Fuzhou, 350002 China

**Keywords:** *Saccharum officinarum*, *Saccharum spontaneum*, LRR-RLK family, Phylogenetic analysis, Expression analysis

## Abstract

**Background:**

Sugarcane (*Saccharum spp*.) holds exceptional global significance as a vital crop, serving as a primary source of sucrose, bioenergy, and various by-products. The optimization of sugarcane breeding by fine-tuning essential traits has become crucial for enhancing crop productivity and stress resilience. Leucine-rich repeat receptor-like kinases *(LRR-RLK*) genes present promising targets for this purpose, as they are involved in various aspects of plant development and defense processes.

**Results:**

Here, we present a detailed overview of phylogeny and expression of 288 (495 alleles) and 312 (1365 alleles) *LRR-RLK* genes from two founding *Saccharum* species, respectively. Phylogenetic analysis categorized these genes into 15 subfamilies, revealing considerable expansion or reduction in certain LRR-type subfamilies. Compared to other plant species, both *Saccharum* species had more significant *LRR-RLK* genes. Examination of cis-acting elements demonstrated that *SsLRR-RLK* and *SoLRR-RLK* genes exhibited no significant difference in the types of elements included, primarily involved in four physiological processes. This suggests a broad conservation of *LRR-RLK* gene function during *Saccharum* evolution. Synteny analysis indicated that all *LRR-RLK* genes in both *Saccharum* species underwent gene duplication, primarily through whole-genome duplication (WGD) or segmental duplication. We identified 28 *LRR-RLK* genes exhibiting novel expression patterns in response to different tissues, gradient development leaves, and circadian rhythm in the two *Saccharum* species. Additionally, *SoLRR-RLK104*, *SoLRR-RLK7*, *SoLRR-RLK113*, and *SsLRR-RLK134* were identified as candidate genes for sugarcane disease defense response regulators through transcriptome data analysis of two disease stresses. This suggests *LRR-RLK* genes of sugarcane involvement in regulating various biological processes, including leaf development, plant morphology, photosynthesis, maintenance of circadian rhythm stability, and defense against sugarcane diseases.

**Conclusions:**

This investigation into gene duplication, functional conservation, and divergence of *LRR-RLK* genes in two founding *Saccharum* species lays the groundwork for a comprehensive genomic analysis of the entire *LRR-RLK* gene family in *Saccharum*. The results reveal *LRR-RLK* gene played a critical role in *Saccharum* adaptation to diverse conditions, offering valuable insights for targeted breeding and precise phenotypic adjustments.

**Supplementary Information:**

The online version contains supplementary material available at 10.1186/s12864-024-10073-z.

## Background

Cells produce receptor proteins to detect extracellular chemical signals elicited by complex mixtures during plant development and defense, which in turn direct downstream cellular responses. Receptor-like protein kinases (RLKs) in plants and receptor tyrosine kinases (RTKs) in animals perform analogous functions in signal transduction by sensing the external environment through their extracellular domains, facilitating effective intercellular communication [1]. Among the RLK superfamily, the leucine-rich repeat receptor-like kinase (LRR-RLK) family constitutes the most abundant subfamily, encompassing the majority of identified *RLK* genes in plants [2]. Typically, a LRR-RLK consists of three functional domains: an extracellular leucine-rich repeat motif (LRR domain) responsible for ligand recognition, a transmembrane domain (TM) anchoring the protein within the cell membrane, and an intracellular kinase domain (KD) responsible for downstream signal transduction through self-phosphorylation [3]. The diversity of LRR domain enables the LRR-RLK to sense a wide array of ligands, including small molecules, peptides, and complete proteins, and the variable amino acids in the conserved region determine the specificity with their ligand interactions [4]. The transmembrane region serves as the connection between the extracellular and intracellular regions, featuring several positively charged basic amino acid residues at its carboxyl terminal, which function as "stop transport" signals. Notably, phosphorylation sites are a prominent characteristic of LRR-RLK protein kinases. Phosphorylation of the kinase domain can activate or enhance the enzyme's activity, and studies have demonstrated that autophosphorylation of specific protein kinases can catalyze their activity in vitro and regulate leaf aging in plants [5]. *LRR-RLK* genes play pivotal roles in various biological processes, including the regulation of plant growth and development, hormone perception, disease defense, and self-incompatibility recognition [6–9]. Their significance as potential targets for crop improvement have made them particularly appealing to researchers [10, 11]. Thus far, the *LRR-RLK* gene family has been extensively identified and characterized in diverse plant species, such as *Oryza sativa* [12], *Arabidopsis thaliana* [13], *Solanum lycopersicum* [14], *Cucumis sativus* [15], and *Glycine max* [16].

Previous studies on *LRR-RLK* gene function have elucidated their two primary biological roles: involvement in plant development and defense against pathogens. For instance, *CLV1* mediated the CLAVATA signaling pathway, regulating meristem development and plant cells division [17]. It collaborates with *CLV2* and *CLV3* to promote plant stem cell differentiation [18]. *AtRUL1* and *AtMOL1* are implicated in the secondary growth of plants [19], while *AtBAM1* and *AtBAM2* play crucial roles in early plant development, cell division and differentiation in *Arabidopsis thaliana* [20]. Simultaneously, *LRR-RLK* genes play a vital role in diverse immune responses in plants. Some *LRR-RLK* genes respond to abiotic and biological stresses. For example, *FLS2* and EFR mediated plant resistance to bacterial pathogens [21]. Certain *LRR-RLK *genes have dual functions in development and defense. For instance, *BAK1* (bri1-associated kinase 1) interacts with phytohormone receptor *BRI1D*, participating in developmental regulatory processes, and also engages with *FLS2*, contributing to innate immunity against pathogens by recognizing bacterial flagellin flg22 peptide [22]. *NIK1* exhibits defensive activity against viral infections and is targeted by nuclear shuttle protein (NSP) of twin viruses, interacting with the NSPs during viral infection [23, 24]. *Xa21* relies on its distinctive LRR motif to recognize toxic substances produced by rice white leaf blight pathogens, triggering the plant's defense response [25].

Sugarcane, a significant sugar and energy crop with global economic and ecological importance. Modern sugarcane cultivar resulting from deliberate interspecific hybridization between *Saccharum officinarum* and *Saccharum spontaneum*. Understanding the functional genomics and molecular biology of these two species can shed light on their contribution to sugarcane hybrid breeding and provide a theoretical basis for molecular improvements in sugarcane breeding. The *LRR-RLK* gene, a prominent plant receptor kinase extensively studied in various plants, has been confirmed to play essential roles in multiple aspects of plant growth and defense processes [17, 26–29]. This underscores the potential of the *LRR-RLK *gene as a promising candidate for enhancing plant traits. However, a comprehensive genome-wide phylogenetic and functional characterization of *LRR-RLK* genes specific to sugarcane is still lacking. To address this gap and facilitate future research on this important gene family, we utilized bioinformatics to identify and classify all *LRR-RLK* gene members from the two founding *Saccharum* species. Additionally, we analyzed the evolutionary relationship and collinearity of these species. Utilizing multiple sets of transcriptome data, we conducted a comprehensive analysis of *LRR-RLK* gene expression patterns in stem and leaf tissues, gradient development leaves, circadian rhythm, and disease-stressed sugarcane leaves. Through this analysis, we explored *LRR-RLK* genes related to sugarcane leaf development, plant morphology, photosynthesis, circadian clock stability, and disease defense, laying a solid foundation for future functional research.

## Results

### Genome-wide identification of *LRR-RLK* genes

**Fig. 1:**
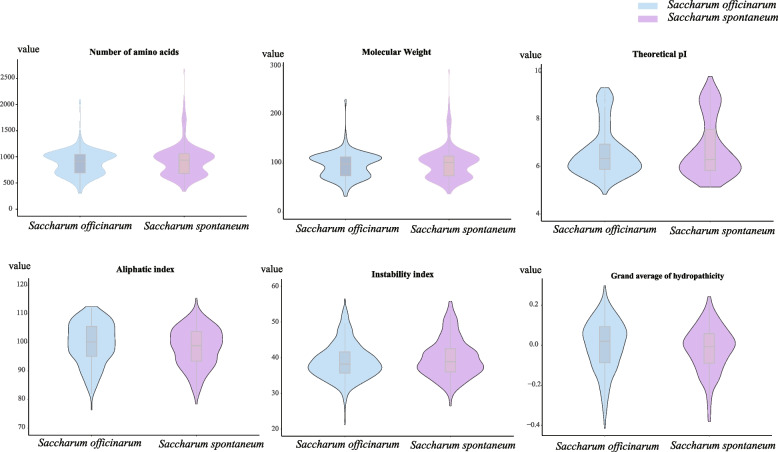
The physical and chemical parameters of LRR-RLK proteins in *Saccharum spontaneum* and *Saccharum officinarum*

A total of 495 and 1365 LRR-RLK protein sequences were identified based on functional annotation (Pfam domains) in the genomes of *S. spontaneum* and *S. officinarum*, respectively (Table 1, Additional file 1). Additionally, 293 Sorghum and 127 Pineapple *LRR-RLK* genes were also identified used same method (Additional file 2). Furthermore, 288 and 312 *LRR-RLK* gens were found in monoploid genome of *S. spontaneum* and *S. officinarum,* respectively (Table 1, Additional file 1), some of which exhibited allelic deletions. The *SsLRR-RLK* and *SoLRR-RLK* genes were named based on their chromosomal locations (Additional file 1). The physicochemical property including amino acid length (NA), molecular weight (NW), isoelectric point (PI), protein instability index (II), aliphatic index (AI), and grand average of hydropathicity (GRAVY) of *Saccharum LRR-RLK* genes were predicted (Fig. 1, Additional file 3). In *S. officinarum*, the NA of LRR-RLK family proteins ranged from 311 to 2094, while NW varied from 32.60 to 230.72 kDa. The pI values were distributed from 4.93 to 9.31. The II index varied from 26.36 to 56.45, while AI ranged from 76.32 to 112.62 and GRAVY from -0.415 to 0.298. According to the rule that the II is less than 40 is a stable protein, and the PI is less than 7 is an acidic protein. Among the 312 haplotypes of *LRR-RLK* genes in *S. officinarum*, 195 were classified as stable acidic proteins, 47 as unstable acidic proteins, and 70 as unstable basic proteins. Additionally, 147 *LRR-RLK* genes showed negative values of GRAVY, while the remaining displayed positive values, with a relatively balanced distribution of both. In *S. spontaneum,* the NA of the 288 SsLRR-RLK proteins varied from 336 to 2678, with relative NW ranged from 37.42 to 292.02. The pI values of the SsLRR-RLK proteins ranged from 5.13 to 10.63, while II and GRAVY is from 26.48 to 55.77 and -0.382 to 0.244, respectively. Of the SsLRR-RLK proteins, 162 were categorized as stable acidic protein, 38 unstable acidic proteins and 88 unstable basic proteins.

**Table 1:** Protein classification according to the presence of diagnostic domains *S. spontaneum, S. officinarum, and S. bicolor* proteomes

Predicted proteins	Plant species		
	***S. officinarum***	***S. spontaneum***	***S. bicolor***
Kinases	9854	3558	1878
LRR Kinases	1580	559	324
TM kinases with LRR (KD)^a^	312(1365)	288(495)	293

^a^The numbers in parenthesis represent the total number of kinase domains identified in the TM kinases with LRR

### Phylogenetic analysis of *LRR-RLK* family

**Fig. 2:**
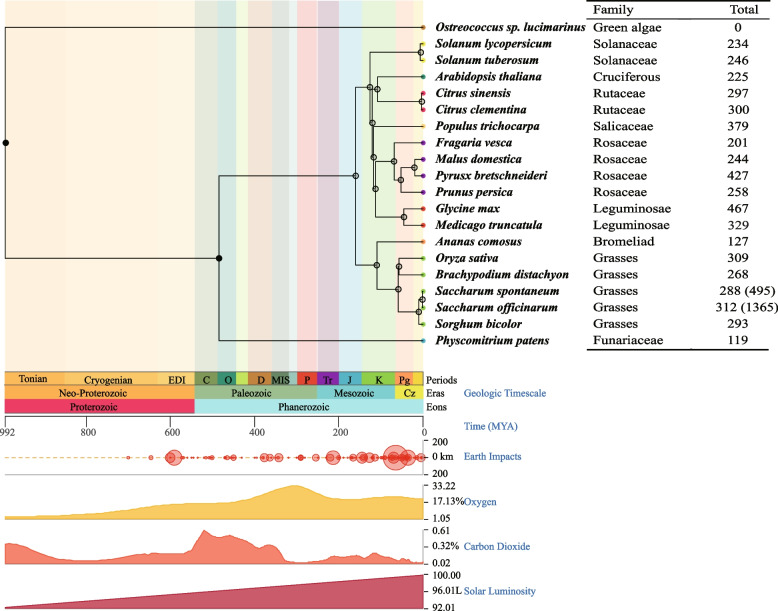
Phylogenetic tree of diverse species showing the number of LRR-RLK family. Linear scale Time Mya (millions of years ago) was shown at the tree’s bottom. 20 plants including *Saccharum spontaneum*, *Saccharum officinarum*, *Oryza sativa*, *Brachypodium distachyon*, *Sorghum bicolor*, *Solanum tuberosum*, *Solanum lycopersicum*, *Arabidopsis thaliana*, *Citrus sinensis*, *Citrus clementina*, *Fragaria vesca*, *Pyrus bretschneideri*, *Malus domestica*, *Prunus persica, Ostreococcus lucimarinus, Physcomitrella patens*, *Populus trichocarpa*, *Medicago truncatula*, *Glycine max, Ananas comosus*

The *LRR-RLK* gene family exhibits a widespread distribution across various plant species. In this study, we conducted a comprehensive analysis by screening 20 representative species from 10 lineages, including Angiosperms such as Undergraduate, Cruciferae, Rosaceae, Solanaceae, and others, as well as lower plants like green dinoflagellates and Physcomitrella plants. Using this dataset, we constructed a species phylogenetic tree to explore the phylogenetic relationships of the LRR-RLK family genes. As depicted in Fig. 2, our analysis reveals a gradual expansion of the LRR-RLK families from algae to terrestrial plants. Notably, lower plant dinoflagellates do not possess any *LRR-RLK* genes, while higher plants exhibit varying numbers of *LRR-RLK* genes. Of particular interest, sugarcane displays a higher number of *LRR-RLK* genes compared to other grass crops. Even after correcting for the haplotype level, sugarcane still outnumbers most species, including well-studied model plants like *Arabidopsis thaliana* and other crops. This observation could be attributed to the polyploid nature of sugarcane, which may contribute to the expansion of the *LRR-RLK* gene family in this crop.

**Fig. 3:**
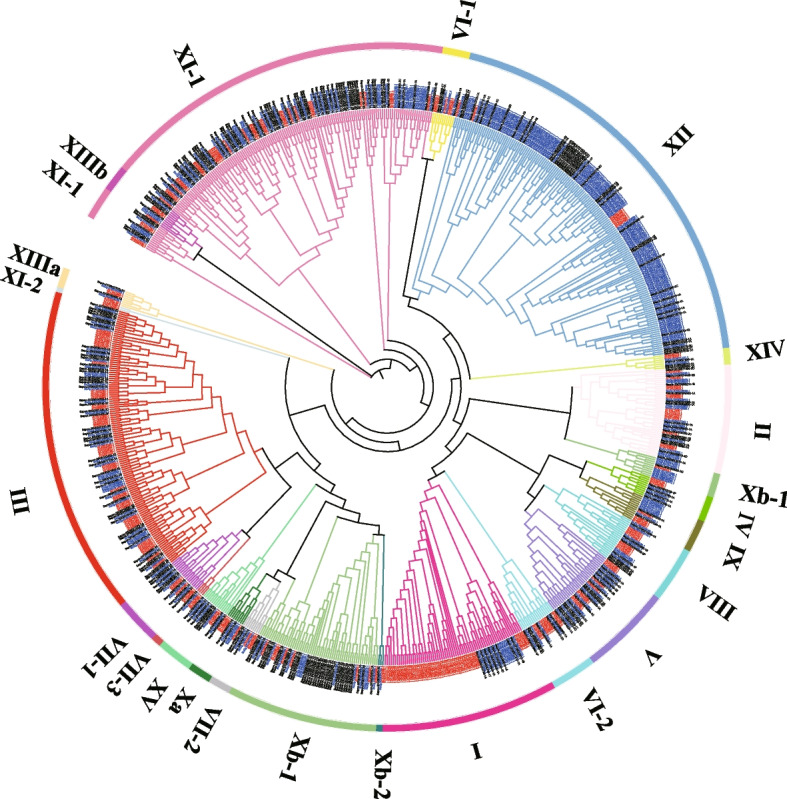
Phylogenetic tree of LRR-RLK from *Arabidopsis thaliana* and two founding *Saccharum* species. A phylogenetic tree of LRR-RLK proteins from *Saccharum spontaneum, Saccharum officinarum,* and *Arabidopsis thaliana* was constructed using FastTree2 with the maximum likelihood method. The two founding *Saccharum* LRR-RLK families were clustered into 15 subgrups. The different colored arcs indicate different groups (or subgroups) of th*e LRR-RLK* genes. The labels marked in black color represent *Saccharum spontaneum*, blue color represent *Saccharum officinarum,* and red color represent *Arabidopsis thaliana*

To gain a deeper insight into the evolutionary relationship of *LRR-RLK* genes in sugarcane, we classified 312 *SoLRR-RLK* and 288 *SsLRR-RLK* gens into 15 subfamilies based on the classification results of *Arabidopsis thaliana* (Fig. 3, Table 2, Additional file 4). Interestingly, the distribution of *LRR-RLK* genes from *Saccharum* species in the tree was not uniform. Notably, the *LRR-RLK* genes of *Arabidopsis thaliana* clustered closely with those of sugarcane. In general, the relative size of each LRR-RLK subclade in sugarcane was almost similar to that of *Arabidopsis thaliana*, with the exception of subclade I and subclade Xb-1, XI-1, and XII (Fig. 3, Table 2). Subclade Xb-1, XI-1, and XII in sugarcane displayed a significantly higher number of *LRR-RLK* genes compared to *Arabidopsis thaliana*, whereas in subclade, the number of *LRR-RLK* genes in sugarcane was significantly lower than that in *Arabidopsis thaliana*. This observation suggests that the expansion of *LRR-RLK* genes in sugarcane was notably pronounced in subfamily Xb-1, XI-1, and XII, indicating distinct evolutionary patterns between monocots and dicots.

**Table 2:** Total number of genes distributed in the LRR-RLK different subgroups

Subgroups	Plants species		
	***A. thaliana***	***S. spontaneum***	***S. officinarum***
LRR-I	50	8	12
LRR-II	14	15	14
LRR-III	46	47	37
LRR-IV	3	4	3
LRR-V	9	15	12
LRR-VI-1	5	3	3
LRR-VI-2	8	6	4
LRR-VII-1	5	8	5
LRR-VII-2	3	3	3
LRR-VII-3	2	1	1
LRR-VIII	8	7	7
LRR-IX	4	2	7
LRR-Xa	4	2	3
LRR-Xb-1	9	39	22
LRR-Xb-2	1	1	1
LRR-XI-1	33	67	54
LRR-XI-2	2	0	0
LRR-XII	8	43	112
LRR-XIIIa	4	2	2
LRR-XIIIb	3	4	3
LRR-XIV	2	3	2
LRR-XV	2	8	5
Total	225	288	312

### Cis-acting elements analysis on promoters of *LRR-RLK* genes

**Fig. 4:**
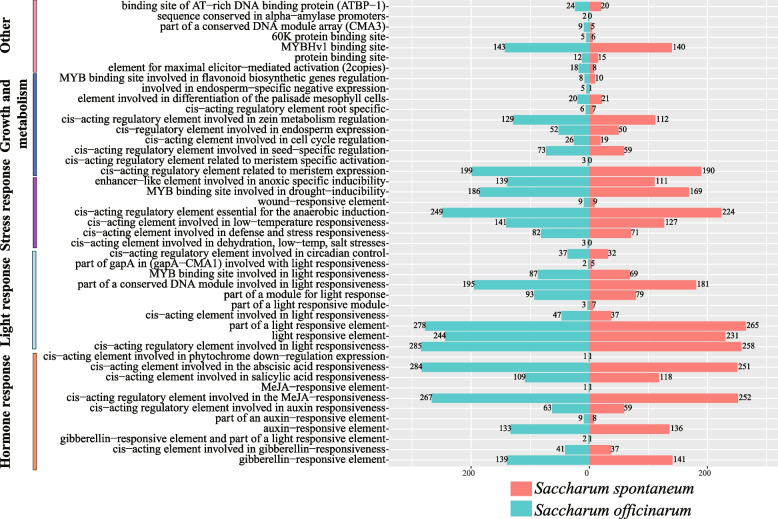
Cis-elements analysis of *LRR-RLK* genes in *Saccharum spontaneum* and* Saccharum officinarum*

Analysis of the cis-acting elements in the promoter region is crucial for understanding gene function, as these elements play a significant role in gene transcription and expression. Functionally of the *LRR-RLK* genes in both *Saccharum* species can be divided into four main categories: light response, phytohormone response, stress induction, and plant growth metabolism (Fig. 4). The first category is photoresponsive elements, with a vast majority of sugarcane *LRR-RLK* genes containing such elements. For example, conserved G-boxes and GT1-motifs are widely present in the upstream sequence of these genes. The second category involves phytohormone-responsiveness elements. Elements associated with the responses to methyl jasmonate (MeJA) and abscisic acid (ABA) were relatively abundant in the *LRR-RLK* genes of both two original sugarcane species, respectively. Specifically, in *S. officinarum* and *S. spontaneum,* 252 and 267 *LRR-RLK* gene promoters were enriched in the cis-acting elements CGTCA motif and TGACG motif, respectively, which are involved in the MeJA response. Moreover, 251 and 284 gene promoters were enriched in ABRE involved, related to the ABA response, respectively. These results suggest that these *LRR-RLK* genes may regulate MeJA and ABA signaling in sugarcane, playing an essential role in plant defense and leaf abscission. The third category is the cis-acting element involved in stress response. The *LRR-RLK* genes contain cis-acting elements associated with hypoxia induction, drought induction and low temperature stress, including LTR, TC-rich repeats, ARE and MBS, etc. These findings indicate that *LRR-RLK* genes may be involved in defense response and stress response. Lastly, the fourth category includes elements responding to plant growth and metabolism, such as GCN4-motif, CAT-box, RY-element, etc. Additionally, specific cis-acting elements like NON-box, A-box and DRE were only found in *SsLRR-RLK* promoters. The analysis of cis-acting elements in the promoter region of the *LRR*-*RLK* genes in both Saccharum species revealed no singnificant differences and indicated their involvement in a diverse range of biological processes. This suggests that the function of *LRR*-*RLK* genes is conserved across various physiological processes in both *Saccharum* species.

### Gene duplications, chromosomal locations, and syntenic relationship of LRR-RLK family genes

**Fig. 5:**
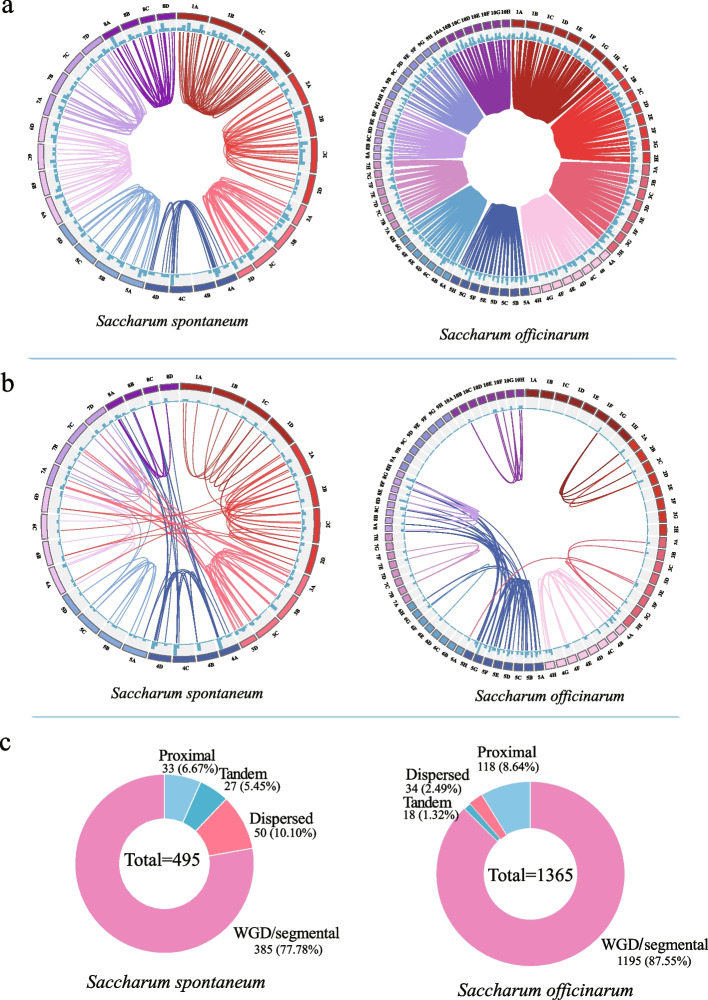
Collinearity relationships of *LRR-RLK* genes on the *Saccharum spontaneum* and *Saccharum officinarum* genome. **a** Synteny analysis of alleles of *LRR-RLK* genes in two founding *Saccharum* species. **b** Synteny analysis of non-alleles of *LRR-RLK* genes in two *Saccharum* species. **c** Number of *LRR-RLK* genes from different origins in two founding *Saccharum* species

Gene duplication is a prominent feature for genomes, and numerous studies have showed that gene families evolve through genome-wide, segmental, or tandem replication, followed by genes diversification [30]. To gain further insight into the amplification mode of the *LRR-RLK* gene family in *Saccharum*, we analyzed gene duplications and syntenic relationship of the complete *LRR-RLK* genes. As illustrated in Table 3, a total of 1365 *SoLRR-RLK* genes and 495 *SsLRR-RLK* genes, including alleles, were unevenly distributed among the 8 homologous genomes of *S. spontaneum* and the 10 homologous genomes of *S. officinarum*, respectively (Table 3). In *S. spontaneum,* the distribution of *SsLRR-RLK* genes among the 8 homologous chromosome groups was relatively similar, with the highest number on chromosome 2 (88, 17.78%), and the lowest number on chromosome7 (46, 9.29%). We identified 305 syntenic pairs of *SsLRR-RLK* genes in *S.spontaneum*, including 186 pairs of alleles and 119 pairs of non-alleles (Fig. 5a and b, Additional file 5). Similarly, in *S. officinarum,* chromosome 2 contained the most *SoLRR-RLK* genes (168, 12.31%), while chromosome 7 had the least (85, 6.23%). A total of 11,987 syntenic gene pairs were identified, of which 1901 were alleles syntenic pairs and 87were non-allelic syntenic pairs (Fig. 5a and b, Additional file 5). To reveal the amplification method of *LRR-RLK* gene family, we categorized the duplication types of all *LRR-RLK* genes (Fig. 5c, Additional file 6). Among the two founding *Saccharum* species, most *LRR-RLK* genes were amplified through WGD or segmental events. Specifically, 385 (77.78%) *SsLRR-RLK* genes and 1195 (87.54%) *SoLRR-RLK* genes originated from WGD or segmental duplications. Additionally, 50 (10.10%), 33 (6.67%), and 27 (5.45%) of *SsLRR-RLK* genes, and 34 (2.49%), 118 (8.64%), and 18 (1.32%) of *SoLRR-RLK* genes were duplicated from dispersed, proximal, and tandem events, respectively. These findings indicated that the sugarcane *LRR-RLK* gene family primarily underwent amplified through gene duplication, with WGD or segmental duplication being the predominant mechanisms.

**Table 3:** The chromosome distribution of *LRR-RLK* genes in sorghum and sugarcane

Chromosome in *S.bicolor*	Number	Chromosome in* S. spontaneum*	Number	*Chromosome in S. officinarum*	Number
SbChr1	30	SsChr1	35(72)	SoChr1	32(164)
SbChr2	46	SsChr2	51(88)	SoChr2	27(168)
SbChr3	42	SsChr3	38(66)	SoChr3	30(167)
SbChr4	35	SsChr4	50(68)	SoChr4	40(158)
SbChr5	29	SsChr5	31(50)	SoChr5	58(160)
SbChr6	20	SsChr6	28(51)	SoChr6	24(98)
SbChr7	20	SsChr7	26(46)	SoChr7	20(85)
SbChr8	22	SsChr8	29(54)	SoChr8	30(112)
SbChr9	24	-	-	SoChr9	21(106)
SbChr10	25	-	-	SoChr10	28(145)

Columns 4 and 6 in the table represent the number of *LRR-RLK* genes distributed on the corresponding chromosomes, and the number of alleles is in parentheses

**Fig. 6:**
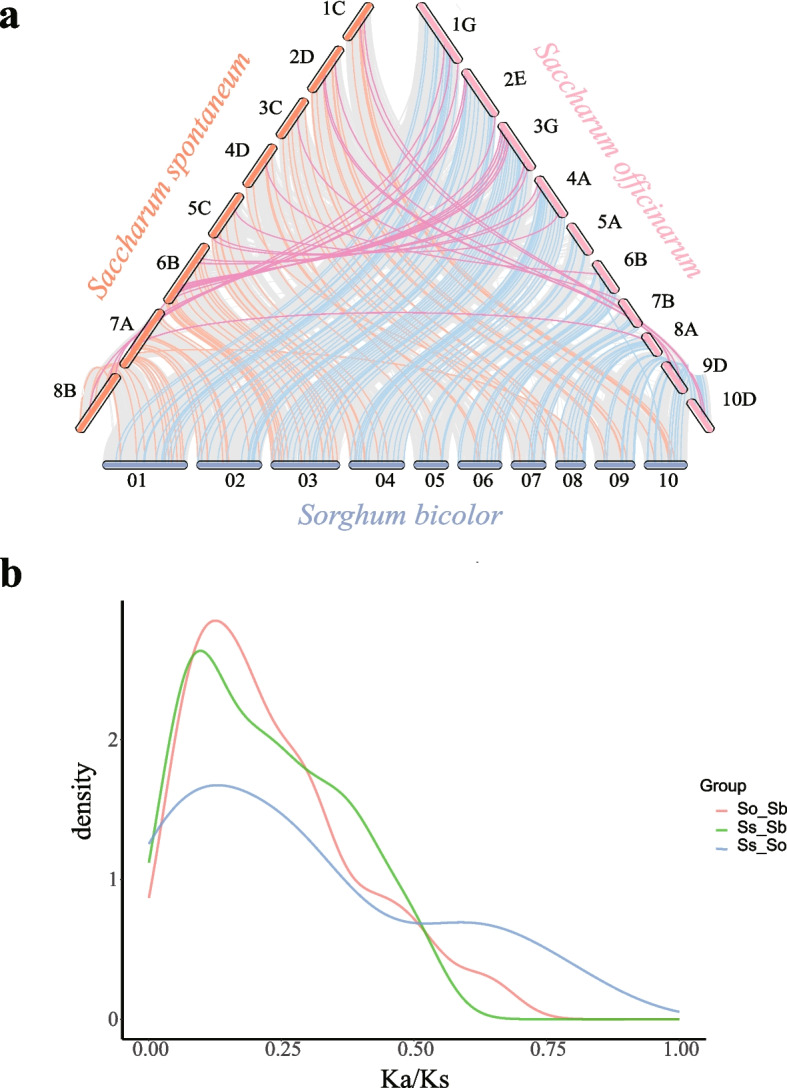
Synteny and Ka/Ks ratio calculated analysis of *LRR-RLK* genes. **a** Synteny analysis of *LRR-RLK* genes from *Sorghum bicolor* with two *Saccharum* species. Gray lines in the background, orange and blue lines between *Saccharum* and *Sorghum bicolor*, and pink lines between *Saccharum spontaneum* and *Saccharum officinarum* indicate the collinear blocks and syntenic LRR-RLK pairs in normal and recombinant regions of chromosomes, respectively. **b** The Ka/Ks ratio of orthologs *LRR-RLK* genes from *Sorghum bicolor and Saccharum*

To further clarify the evolutionary mechanism of *LRR-RLK* genes in *Saccharum*, we constructed an among-species syntenic map of the *Saccharum* single chromosome set and *Sorghum bicolor*, as shown in Fig. 6a. We identified 118 and 71 orthologous *LRR-RLK* gene pairs between the two *Saccharum* species (*S. officinarum* and *S. spontaneum*) and *Sorghum bicolor*, respectively, and 26 pairs in *S. officinarum* and *S. spontaneum* homologous pairs (Fig. 6a). Furthermore, we calculated the ratio of Ka/Ks for the randomly selected orthologous *LRR-RLK* gene pairs (Fig. 6b). The results indicated that all the Ka/Ks ratios were less than 1.0, and the Ka/Ks ratio of different species is most concentrated between 0 and 0.5, implying that purifying selection likely played a dominant role in driving the evolution of the *LRR-RLK* gene family in both *Saccharum* and *sorghum* since their divergence. We estimated the divergence time between the distributed *SsLRR-RLK* and *SoLRR-RLK* genes and their orthologous *SbLRR-RLK* genes based on the pairwise Ks values (Additional file 7). According to previous reported, *S. spontaneum* diverged from the common ancestor of *S. spontaneum* and *S. bicolor* approximately 7.779 million years ago (MYA) [31]. The estimated divergence times between *SsLRR-RLK* genes and their orthologous *SbLRR-RLK* genes ranged from 2.910 to 41.831 MYA, and for *SoLRR-RLK* genes and their orthologous *SbLRR-RLK* genes, it ranged from 2.709 to 103.938 MYA. Additionally, the divergence times between *SsLRR-RLK* and *SoLRR-RLK* orthologs genes were estimated to be 0.609 to 11.676 MYA. Notably, a significant proportion 70.42% (50 genes) for *Saccharum* species and 66.95% (79 genes) for *S. bicolor* exhibited divergence times between 8.00 to 41.831 MYA and 7.924 to 103.938 MYA, respectively. These extended divergence times suggest the antiquity of the LRR-RLK family. Moreover, among the 26 orthologous gene pairs between *S. officinarum* and *S. spontaneum*, 22 pairs displayed a shorter divergence time of 0.609 to 5.658 MYA, underscoring a comparatively later divergence within th*e Saccharum* species than from *S. bicolor* and *S. spontaneum*.

### The expression patterns of *LRR-RLK* genes at different tissues, gradient developmental leaf segments, and circadian rhythm in *Saccharum*

**Fig. 7:**
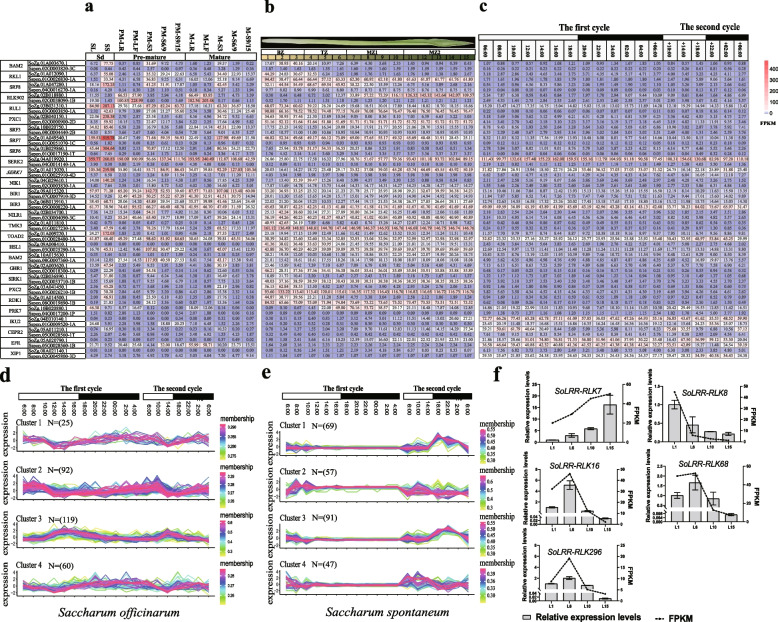
Expression of *LRR-RLK* genes in different tissues, leaf gradients, and day-night rhythms in *Saccharum.* **a** The expression heatmap of *LRR-RLK* genes in different tissues of two *Saccharum* species. **b** The expression heatmap of *LRR-RLK* genes in leaf gradients of two *Saccharum* species.** c** The expression pattern of *LRR-RLK* genes in circadian rhythms in two *Saccharum* species. **d** The Clustering of C-means expression trend of *LRR-RLK* gene family in circadian rhythms from *Saccharum officinarum*, which has four types in total. **e** The Clustering of C-means expression trend of *LRR-RLK* gene family in circadian rhythms from *Saccharum spontaneum*, which has four types in total. **f** RT-qPCR verification the expression pattern of *SoLRR-RLK* genes in gradient developmental leaves. Sd: Seedling stage; PM: Pre-mature; M: Mature; SL: Seeding leaf; SS: Seeding stem; LR: Leaf roll; LF: Leaf; BZ: Basal zone; TZ: Translational zone; MZ1: Maturing zone 1; MZ2: Maturing zone 2

To characterize the expression and physiological function for *LRR-RLK* genes, we performed RNA-seq analysis on different tissues and developmental stage of two founding *Saccharum* species, totaling 12 samples (Fig. 7, Additional file 8 and 9). By analyzing the expression patterns of all *LRR-RLK* gene members in different tissues and developmental stages of *Saccharum,* we observed two distinct expression trends: high expression in stem tissues and high expression in leaf tissues (Additional file 11). The first expression trend includes *LRR-RLK* genes from Cluster1 and Cluster4, which show highly expressed in stem tissues of both *Saccharum* species. This suggests that the *LRR-RLK* genes primarily play roles in *Saccharum* stem growth and sugar metabolism. Moreover, these stem-associated genes can be further classified into two types: those predominantly active during the young stem period and those predominantly active during the mature stem period. The second expression trend involves *LRR-RLK* genes from Cluster2 and Cluster3, which exhibit high expression levels in *Saccharum* leaf tissues at different developmental stages. This indicates that these *LRR-RLK* genes are mainly involved in the regulation of processes within sugarcane leaves. Similar to the stem-associated genes, the leaf-associated genes can also be divided into two types: those highly expressed during the early leaf stage and those highly expressed during the mature leaf stage. The expression profiles of the *LRR-RLK* genes in the two *Saccharum* species showed that approximately 279 (96.88%) *SsLRR-RLK* and 294 (94.23%) *SoLRR-RLK* genes were expressed (FPKM > 0) in all leaf and stem tissues (Fig. 7a, Additional file 8 and 9). Among these, some genes exhibited high expression levels in all three developmental stages of sugarcane leaves but low expression in stem tissues. Notably, genes like *SERK1*, *SERK2*, *PRK7*, and *RLK902* were highly expressed in sugarcane leaf tissues, suggesting their potential involvement in leaf-related processes. Conversely, some *LRR-RLK* genes demonstrated significantly highly expression in stem tissues of two *Saccharum* species, with limited expression in leaf tissues. Examples include *RUL1*, *RLK1*, *SRF6*, *SRF7*, *SRF8*, and *NIK1*, implying that these genes are more likely to play roles in sugarcane stem growth and development. Additionally, we observed differential expression of certain genes between the two founding *Sacchrum* species, such as *BIR1*, *BIR3*, *TOAD2*, and *HLS2*, indicating potential variations in their functions or regulatory mechanisms from two founding *Sacchrum* species.

To gain further insight into the functional divergence of *LRR-RLK* genes in sugarcane leaf morphogenesis and photosynthesis between the two *Saccharum* species, we conducted an expression profile analysis of *LRR-RLK* genes along a continuous leaf developmental gradient (Fig. 7b, Additional file 8 and 9). In two *Saccharum* species, 6 (2.10%) *SsLRR-RLK* and 16 (5.12%) *SoLRR-RLK* genes showed no detectable transcripts, respectively (Additional file 8 and 9). Cluster analysis of the expression patterns of all *LRR-RLK* genes in the leaves of *S. officinarum* revealed two main trends: some *LRR-RLK* genes showed expression patterns that were positively correlated with leaf developmental gradients, while others showed a negative correlation (Additional file 11). Specifically, *SoLRR-RLK* genes from Cluster2 and Cluster4 exhibited a positive correlation with leaf developmental gradients, suggesting their involvement in C4 photosynthesis co-expression regulatory network in sugarcane leaves. Additionally, these genes may play roles in various biological processes related to chlorophyll synthesis metabolism, light reactions, the Calvin cycle, and carbohydrate metabolism in the leaf. On the contrary, *SoLRR-RLK* genes from Cluster1 and Cluster3 showed a negative correlation with leaf developmental gradients, suggesting their regulatory roles in sugarcane leaf cell division, differentiation, and growth hormone metabolism, among other processes. Interestingly, the clustering of expression patterns in the *S. spontaneum* differed from that of the *S. officinarum* (Additional file 11). For instance, *SsLRR-RLK* genes from Cluster1 showed no expression across all leaves developmental gradients of the *S. spontaneum*, while *SsLRR-RLK* genes from Cluster2 and Cluster3 exhibited the highest expression levels at the base of the leaves. Moreover, the transcript abundance of certain genes gradually decreased with increasing leaf maturity (from basal to tip) (Fig. 7b, Additional file 8 and 9). For example, *RLK1*, *GR1, SIRK1*, *TMK3*, and *RDK1* showed such a trend, suggesting their potential roles in leaf growth and development. Furthermore, some *LRR-RLK* genes displayed peak expression levels in the tip region of the leaves, particularly *SERK2*, *SERK1* and *RLK902*. These findings indicate that these genes may be involved in the establishment of photosynthetic organelles in sugarcane leaves. However, certain genes exhibited divergent expression patterns in the gradient developmental leaves of the two sugarcane plants, suggesting potential functional differentiation between them. For instance, genes like *TMK3, PXC2,* and *RDK1* displayed contrasting expression trends. To validate these findings, we performed quantitative real-time PCR (RT-qPCR) experiments on a subset of these genes in three leaf segments of *S. officinarum* (Fig. 7f). The results corroborated the expression patterns observed in the transcriptome data.

The plant's biological clock is one of the most important means of controlling the various life activities of the organism. Plant genes exhibit different expression during circadian rhythm changes and exploring the relationship between sugarcane *LRR-RLK* genes and photosynthesis, as well as the stability of the biological clock, can be explored through their expression patterns during night and day. To investigate the major expression patterns of the LRR-RLK family in circadian biological processes in sugarcane, we analyzed transcriptome data at 19 different time points (Sampling at 2 h and 4 h intervals over two days) in two founding *Saccharum* species, respectively (Fig. 7c, Additional file 8 and 9). In LRR-RLK families, 24 (8.33%) *SsLRR-RLK* genes and 16 (5.12%) *SoLRR-RLK* genes showed undetectable transcription at all-time points, suggesting that these genes do not respond to changes in sugarcane circadian rhythms (Fig. 7c, Additional file 8 and 9). In addition, the *LRR-RLK* gene family showed differential expression trends in the circadian rhythms of two *Saccharum* species (Fig. 7d and e). Specifically, the *LRR-RLK* genes from Cluster1 and Cluster4 were expressed at higher levels in the dark period than during the daytime, with the lowest expression observed at midday when the light intensity was the strongest. This suggests that these genes respond to the day-night transition and are negatively correlated with light intensity. In contrast, the expression levels of *LRR-RLK* genes from Cluster2 and Cluster3 were significantly higher during the daytime than at night, with the gene expression increasing with higher light intensity. This indicates that these genes may be involved in the process of sugarcane leaf photosynthetic response and are positively correlation with light intensity. Consistently, genes such as *BIR3*, *IKU2* and *ERF* showed constitutive expression at all-time points (Fig. 7c, Additional file 8 and 9). Moreover, we observed that some *LRR-RLK* genes displayed different circadian rhythm characteristics in two *Saccharum* species. Notably, *SERK1*, *PRK7*, *TOAD2*, and *XIP1* exhibited distinct expression patterns between the species.

### The Expression patterns of *LRR-RLK* genes under SCMV and PBD infected in *Saccharum*

**Fig. 8:**
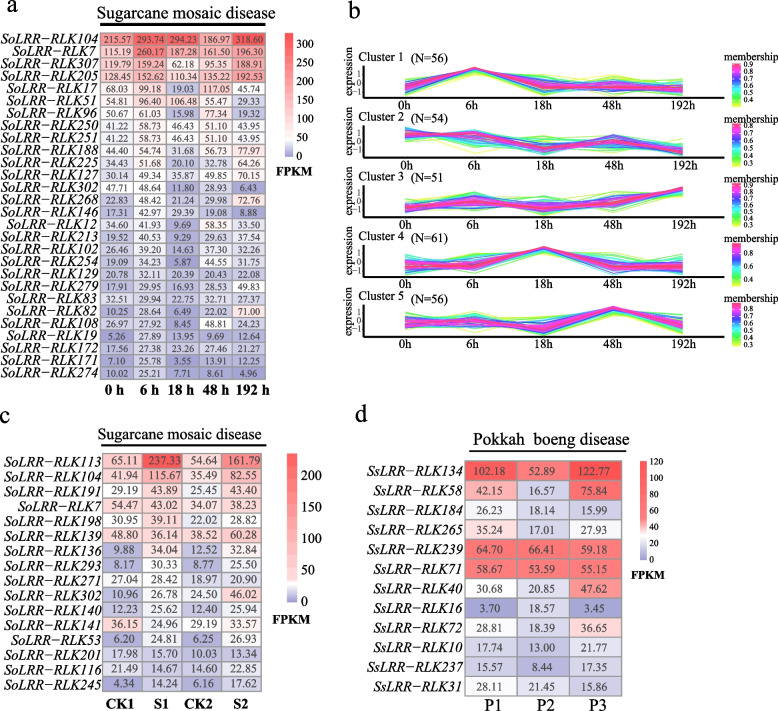
Expression of *LRR-RLK* genes in *Saccharum* infected with different disease. **a** The expression heatmap of *SoLRR-RLK* genes at different time points under SCMV infection. **b** The Clustering of C-means expression trend of *SoLRR-RLK* gene family in *Saccharum officinarum* under SCMV infection, which has five types in total. **c** Expression heatmap of *SoLRR-RLK genes* in different leaves infected with SCMV. **d** Expression heatmap of *SsLRR-RLK* genes in *Saccharum* infected with pokkah boeng disease. P1: CK; P2: inchoate; P3: advanced. CK1: + 1 leaf control; CK2: -3 leaf control; S1: + 1 leaf infection; S2: -3 leaf infection

Sugarcane mosaic disease is a prevalent and highly transmissible viral disease that affects sugarcane [32]. The causative agent, Sugarcane mosaic virus (SCMV), belongs to the Potato Y virus family and can inhibit photosynthesis in sugarcane leaves by damaging chloroplasts, leading to reduced yields. It can also infect other grass crops such as maize and sorghum, as well as some weed species [33, 34]. To investigate the expression patterns of sugarcane *LRR-RLK* genes under virus stress, we analyzed their expression in response to SCMV infestation using two sets of transcriptome data from different time points and different leaves after SCMV infestation (Fig. 8a, Additional file 10). We identified four distinct expression Clusters (Cluster1-Cluster4) of *LRR-RLK* genes in response to SCMV infestation (Fig. 8a and b, Additional file 10). In Clusetr1 and Cluster2, these genes showed the highest expression levels at 6 h after SCMV infestation, indicating a rapid response to viral stress. Examples include *SoLRR-RLK7*, *SoLRR-RLK250*, and *SoLRR-RLK146* suggesting their involvement in the early stress response to disease in sugarcane. The genes in Cluster2 reached their peak expression levels at 192 h after SCMV infestation, with notable examples being *SoLRR-RLK205, SoLRR-RLK307*, and *SoLRR-RLK188,* suggesting their function when viral levels have accumulated over time. On the other hand, in Cluster3 and Cluster4, these genes reached their highest expression levels at 18 h and 48 h after SCMV infestation, respectively, indicating their role in response to viral replication. Notable examples include *SoLRR-RLK51* and *SoLRR-RLK140*. Furthermore, analysis of transcriptome data from different leaves of SCMV-infested sugarcane revealed two main distinct expression trends (Fig. 8c, Additional file 10 and 12). In Cluster1 and Cluster4, both leaves showed a significant increase in gene expression in response to disease stress, with examples such as *SoLRR-RLK113*, *SoLRR-RLK104*, *SoLRR-RLK191* and *SoLRR-RLK293*. Conversely, in Cluster2 and Cluster3, both leaves showed significantly decreased expression levels upon SCMV virus stress, with examples such as *SoLRR-RLK139*, *SoLRR-RLK141*, and *SoLRR-RLK213*. This suggests their involvement in the negative regulatory response of sugarcane to virus-induced pathologies. Notably, *SoLRR-RLK113* and *SoLRR-RLK104* displayed a significantly higher gradient of increasing expression levels in + 1 leaves compared to -3 leaves during virus stress, suggesting a stronger and more pronounced defense response in the fresher leaves of sugarcane.

Pokkah boeng disease of sugarcane (PBD) is a devastating fungal diseases that leads to top rot and eventual top death of sugarcane [35, 36]. To understand the expression patterns of *LRR-RLK* genes in response to varying degrees of PBD infection, we investigated three different expression trends, namely Cluster1-Cluster3, in sugarcane leaves (Fig. 8d, Additional file 10 and 12). In Cluster1, the genes *SsLRR-RLK16*, and *SsLRR-RLK53* showed the highest expression in mildly susceptible leaves. In contrast, the genes *SsLRR-RLK134*, *SsLRR-RLK58*, *SsLRR-RLK72* and *SsLRR-RLK58* in Cluster2 exhibited the highest expression levels in severely susceptible sugarcane leaves. In addition, *SsLRR-RLK239* and *SsLRR-RLK71* were found to be constitutively expressed in both healthy and susceptible leaves. Based on these findings, it can be inferred that the *SsLRR-RLK* genes may play crucial roles in the defense response to PBD in sugarcane. The differential expression patterns of these genes in response to varying degrees of PBD infection suggest their involvement in the plant's defense mechanisms against the fungal pathogen.

## Discussion

*LRR-RLK* genes are widespread in higher plants and play crucial roles in plant various biological processes. However, their systematic study in sugarcane has been limited, making them promising targets for sugarcane breeding and improvement. In plant autopolyploid genomes, genes located at the same position on homologous chromosomes are considered alleles [37]. In this study, we identified, for the first time, 495 and 1365 *LRR-RLK* genes from the genomes of *S. spontaneum and S. officinarum*, respectively, and 288 and 312 haplotype genes, all of which possess the typical LRR extracellular domain, intracellular kinase domain, and transmembrane structure, consistent with the typical characteristics of the LRR-RLK family (Table 1, Additional file 1). Many LRR-domain function in multiprotein complexes [24, 38]. To investigate the evolutionary patterns of *LRR-RLK* genes, we constructed a phylogenetic tree using 20 representative species from 10 families (Fig. 2). The evolutionary trend of the *LRR-RLK* gene family is in line with previous research, showing a sequence from simple unicellular organisms to complex multicellular organisms [4]. Accumulated over time, WGD have likely contributed to gene differentiation and species formation, leading to an increase in the number of *LRR-RLK* genes during plant evolution [39]. Compared to other grass crops, sugarcane *LRR-RLK* genes have undergone significantly expansion, which may be attributed not only to the autopolyploidy nature of its chromosome but also to the impact of allopolyploidization, autopolyploidization, or two rounds of WGD [37, 40]. Previous studies have well-documented the phylogenetic tree of the *LRR-RLK* gene family [41, 42]. According to the LRR-RLK domain feature and clustering relationship of *Arabidopsis thaliana*, the *LRR-RLK* genes in two founding *Saccharum* species were classified into 15 subfamilies (Fig. 3, Table 2). Phylogenetic analysis revealed significant gene amplification in subfamily Xb-1, XI-1, and XII, while the number was significantly reduced in subfamily I. This suggests that subfamilies Xb-1, XI-1, XII and I may have diverged after the split of dicots and monocots.

Cis-acting elements are essential nucleotide sequences located upstream of genes that bind to transcription factors and regulate gene expression and transcription [43]. In two founding *Saccharum* species, the cis-acting elements of the LRR-RLK family are mainly involved in four physiological processes: light response, phytohormone response, stress induction, and plant growth metabolism (Fig. 4). The number and types of cis-acting elements in the *LRR-RLK* genes of two *Saccharum* species are mostly similar, suggesting that these genes are functionally conserved during the evolutionary process. However, there are some cis-acting elements specific to *SoLRR-RLK* genes, indicating that the *SoLRR-RLK* genes may have acquired new functions during replication. Overall, the research findings indicate that the sugarcane *LRR-RLK* genes exhibit a broader spectrum of biological functions. Gene duplication is a major mechanism leading to gene amplification and functional divergence [44, 45]. Previous research has reported that the expansion of the *LRR-RLK* gene family is attributed to gene duplication events, such as WGD, segmental duplication, and tandem duplication [4, 41, 42, 46]. Whole-genome duplication, segmental duplication and tandem duplication are the three most common types of gene duplication in plants, and polyploid plants retain many duplicated blocks of chromosomes in their own genomes after chromosomal rearrangements [47–49]. In this study, we analyzed the duplication patterns of *LRR-RLK *genes in two founding *Saccharum* species (Fig. 5, Additional file 6). Approximately 385 (77.78%) *SsLRR-RLK g*enes and 1195 (87.54%) *SoLRR-RLK* genes were amplified from WGD or segmental events, respectively. Likewise, we investigated the synteny relationship between the two founding *Saccharum* species and sorghum and identified 118 and 71 pairs of LRR-RLK paralogous gene pairs, respectively (Fig. 6a). The ratio of Ka/Ks between the randomly selected orthologous *LRR-RLK* genes were found to be less than 1.0, indicating that these paralogous genes have undergone strong purifying selection for retention (Fig. 6b). Thus, Sugarcane and Sorghum may be strongly driven by purification selection during their evolutionary differentiation.

The potential function of genes can be inferred from their expression levels in various aspects of the plant [50]. In this study, with the aim of exploring the potential functions of *LRR-RLK* genes in *Saccharum* species, we investigated *LRR-RLK* genes expression patterns based on six sets of RNA-seq data (Fig. 7 and 8). Previous studies had reported that the involvement of *AtBRL1* and *AtBRL3* in the regulation of plant growth and development [51]. *MOL1* and *RUL1* as opposing regulators of secondary growth [52]. *RLK1* plays an important role in pear pollen tube elongation and cell wall integrity [53]. In our research, we identified some *LRR-RLK* genes that were specially expressed in stem tissues of the *Saccharum* species (Fig. 7a, Additional file 8 and 9), such as *RUL1*, *RLK1*, *SRF6*, *SRF7*, *SRF8*, indicating that these *LRR-RLK* genes might regulate biological processes related to stem development. Conversely, *SERK2* and *RLK902* showed higher expression levels in leaves than in stems, indicating their tissue-specific function, mainly function in leaf tissues (Fig. 7a). This is consistent with previous reports that *AtCLV1*, *AtCLV2*, and *AtCLV3* jointly promote plant stem cell division, thereby regulating plant growth and development [18]. These genes may play a role in the contemporary breeding research of sugarcane, particularly in the regulation of plant architecture. In graminaceous monocots, leaf development and photosynthetic differentiation follow a pattern of persistence and height from leaf base to leaf tip, with the leaf base region enriched in basal cell functions, such as DNA synthesis, synthesis of primary cell walls, and signaling of hormones such as auxin [54–56]. Our current study showed that the expressions of *RLK1*, *GR1*, *SIRK1*, *TMK3*, and *RDK1*, among others, were higher in the basal region of leaves, suggesting their involvement in hormone signaling and cell wall biosynthesis in leaf primary metabolism (Fig. 7b, Additional file 8 and 9). On the other hand, *SERK1*, *SERK2*, and *RLK902* were highly expressed in the tip regions of the leaf, indicating that their potential associated with photosynthesis. Gene expression levels change with circadian rhythms, enabling plants to coordinate their metabolism and development in response to environmental changes [57, 58]. Previous studies have showed that plants gene expression levels vary in response to changes in circadian rhythms and certain periodicity [59, 60]. Our findings indicate that the sugarcane *LRR-RLK* gene family exhibits significantly different expression trends during circadian rhythm changes. *SERK1*, *PRK7*, *TOAD2*, and *XIP1* showed different expressions between the two *Saccharum* species (Fig. 7c, Additional file 8, 9, and 11). Conversely, *BIR3*, *IKU2*, and *ERF* did not respond to changes in circadian rhythms, suggesting that their potential role in maintaining the stability of the sugarcane circadian clock (Fig. 7c, Additional file 8 and 9). In summary, the *LRR-RLK* genes expression profile obtained in this study serves as a crucial reference for delving further into the functionalities of *LRR-RLK *genes in sugarcane. It also introduces novel avenues for regulating growth and development, plant morphology, photosynthesis, and circadian clock stability in the realm of sugarcane breeding research. These findings open new horizons for the identification of potential candidate genes.

Participating in the disease defense response process is an important function of *LRR-RLK* genes, and they are extensively involved in defense against pathogens [10, 61, 62]. In *Arabidopsis thaliana*, *AtNIK1*, *AtNIK2*, and *AtNIK3* have been reported to interact with NSPs and participate in virus defense [63]. In our study, we identified several defense-related LRR-RLK candidate genes against SCMV, such as *SoLRR-RLK04, SoLRR-RLK205, SoLRR-RLK113*, and *SoLRR-RLK104* (Fig. 8a and c, Additional file 10). These genes showed up-regulated or down-regulated expression levels to varying degrees upon SCMV infection. On the other hand, some genes, such as *SoLRR-RLK118*, *SoLRR-RLK106*, *and SoLRR-RLK83,* exhibited stable expression levels after SCMV infection, suggesting that they may not respond to virus infection (Fig. 8a and c). Likewise, *LRR-RLK* genes have been found to confer resistance to pathogenic bacteria. For example, *OsSERK1-2* in rice is activated by pathogen signals or other stress signals to regulate immune signaling pathways [64, 65]. *BAK1/SERK3* has also been shown to play roles in the immune response against the late blight pathogen (Phytophthora infestans) [65, 66]. In our study, certain *SsLRR-RLK* genes, such as *SsLRR-RLK16, SsLRR-RLK53, SsLRR-RLK134*, *SsLRR-RLK40*, *SsLRR-RLK72*, and *SsLRR-RLK58*, were identified as differentially expressed in sugarcane leaves with mild or severe PBD (Fig. 8d, Additional file 10). Numerous candidate genes associated with defense mechanisms, particularly those pertaining to pokkah boeng disease and mosaic disease in sugarcane, have been discerned as putative *LRR-RLK* genes. This identification underscores the pivotal role of *LRR-RLK* genes as integral components within plant defense mechanisms, specifically in countering fungal and viral pathogens. These findings provide valuable insights into the sugarcane disease resistance mechanism.

## Conclusions

In this study, we conducted a comprehensive analysis of putative *LRR-RLK* genes in the genomes of *S. spontaneum* and *S. officinarum*, revealing 288 (495 alleles) and 312 (1365 alleles) genes, respectively, which were clustered into 15 distinct subfamilies. Notably, the Xb-1, XI-1, and XII subfamily exhibited a higher gene count compared to other subfamilies in both *Saccharum* species, while the I subfamily displayed a significant reduction in gene numbers. Cis-element analysis highlighted the functional conservation of *LRR-RLK* genes in two founding *Saccharum* species, with their roles spanning various facets of plant growth. Our investigation identified gene expansion events in the two *Saccharum* species, likely attributed to gene duplication processes, predominantly through WGD or segmental duplication events. Furthermore, by assessing the Ka/Ks ratio, we deduced that the differentiation between sugarcane and sorghum was driven by robust purification selection. Through transcriptome data analysis, we elucidated the potential functions of *LRR-RLK* genes in diverse biological processes within sugarcane. Specifically, genes such as *RLK1*, *GR1*, and *SIRK1* exhibited tissue-specific expression patterns and were implicated in leaf growth and development. Meanwhile, *BIR3, IKU2*, and *ERF* genes were postulated to contribute to the maintenance of the sugarcane circadian clock's stability. Several genes, including *SoLRR-RLK113*, *SoLRR-RLK104*, *SoLRR-RLK7*, *SoLRR-RLK293*, *SsLRR-RLK134,* and *SsLRR-RLK58*, were identified as potential players in the defense response against sugarcane diseases. Moreover, differential expression was observed in genes such as *TOAD2*, *XIP1*, *BIR1*, and *HLS2* between the two *Saccharum* species, suggesting the possibility of functional differentiation. This comprehensive dataset not only provides valuable insights into the roles of *LRR-RLK* genes and their molecular mechanisms governing development, plant morphology, photosynthesis, circadian rhythm stability, and defense responses in *Saccharum*, but also offers a promising direction for molecular breeding strategies in this important crop.

## Materials and methods

### Plant materials

The sugarcane materials used in this study were LA-purple (*S. officinarum*, 2n = 8x = 80) and SES 208 (*S. spontaneum*, 2n = 8x = 64) were planted in the Greenhouse of Fujian Agriculture and Forestry University (Fuzhou, China), Fuguo 1 (*S. officinarum*, 2n = 8x = 80) which were planted in the Specimen Garden of Guangxi University (Nanning, China). For the three different developmental stages (Seedling, pre-maturity, and maturity) experiment, stem and leaf tissue samples from the two founding *Saccharum* species were obtained as described by previously described [67, 68]. To analyze expression patterns during leaf development, the gradient developmental leaf experiments was conducted following the method described by Hu et al. Specifically, 15 cm leaves were selected and cut into 15 segments, each one centimeter in length [54, 69]. To analyze circadian rhythm characteristics, sugarcane leaf samples were collected at 19 different time points, sampling at 2 h and 4 h intervals over two days, as described in previous research [70]. For the SCMV infection experiment, + 1 leaves of 4 different periods (infected with 6 h, 18 h, 48 h and 192 h) were taken, with 0 h control plants not infected. In addition, sugarcane + 1 leaves and -3 leaves were selected as the infection objects, and the leaves were collected one month after the infestation, and the uninfected leaves were used as a control (CK) [71]. RNA-seq for PBD was extracted from modern hybrid sugarcane ZZ1. The mildly diseased leaves and severely diseased leaves were selected for analysis, while healthy leaves were used as control (CK) [71].

### RNA extract and RT-qPCR

Total RNA from sugarcane gradient development leaf material was isolated using Trizol (Invitrogen) reagent kit for each sample. Subsequently, cDNA was synthesized using the reverse transcription kit StarScript II First-strand cDNA Synthesis Kit With gDNA Remover (A222-10, Genstar). Primer design, RT-qPCR reaction procedure, and instrument used for RT-qPCR were referenced from Li et al. [72]. The RT-qPCR amplification was performed using 2 × RealStar Green Fast Mixture (A301–10, Genstar) on a Multicolor Real-Time PCR Detection System (Bio-Rad). The reaction program followed the two-step method outlined in the kit's protocol: initial denaturation at 95 °C for 2 min, followed by 40 cycles of denaturation at 95 °C for 15 s and annealing/extension at 60 °C for 30 s. The relative expression of the target genes was calculated using the 2^−ΔΔCt^ method [73], with GAPDH serving as the reference gene [74]. And graphical representation were performed using GraphPad Prism 9.0 software [75]. The gene-specific primers used for RT-qPCR are listed in Additional file 13.

### Identification of *LRR-RLK* gene family in the *Saccharum*

The genomic data of the two *Saccharum* species, *S. spontaneum* and *S. officinarum*, were generated in our laboratory [37]. Hidden Markov model (HMM) profiles (PF 00069 and PF 07714) downloaded from the Pfam database (http://pfam.xfam.org/) as search models for kinase structure. The following LRR diagnostic domains were searched: LRR_1 (PF00560), LRRNT (PF 01462), LRV (PF 01816), LRRNT_2 (PF 08263), LRR_4 (PF 12799), LRR_5 (PF 13306), LRR_8 (PF 13855), LRR_9 (PF 14580), LRRCT (PF 01463), LRR_2 (PF 07723), LRR_3 (PF 07725) [76]. The HMMsearch tool was used to search for LRR-RLK family members from local protein database of the tow *Saccharum* species (*S. spontaneum* and *S. officinarum*). In addition, 225 known LRR-RLK proteins sequences in the *Arabidopsis thaliana* were used as query sequences. we conducted a thorough comparison using the BLAST software to eliminate duplicate sequences and those with incomplete structural domains [77]. By combining these two methods, the *LRR-RLK* genes for *S. spontaneum* and *S. officinarum* was tentatively identified.

The conserved domain of the candidate gene proteins was predicted using two online tools: SMART (http://smart.emblheidelberg.de/) and NCBI CD-search (https://www.ncbi.nlm.nih.gov/Structure/cdd/wrpsb.cgi). The genes with the same protein structure as *Arabidopsis thaliana* LRR-RLK were further screened. Transmembrane helices were predicted using TMHMM website (http://www.cbs.dtu.dk/services/TMHMM/). Only candidate proteins containing the extracellular LRR domain, the transmembrane region, and the intracellular serine/threonine kinase domain were considered putative LRR-RLK genes. Sorghum and pineapple protein sequences were downloaded from the Phytozome (https://phytozome.jgi.doe.gov/) and EnsemblPlant (http://plants.ensembl.org/index.htmL) plant genome websites, respectively. Their LRR-RLK family members were identified by the same method as *Saccharum*. Using the allele table provided by our own laboratory, the LRR-RLK alleles of the two *Saccharum* species were distinguished and a single set of *LRR-RLK* genes was screened out. In *S. officinarum*, the two contigs that are not mounted on the chromosome are regarded as a single gene. The haplotype genes of the LRR-RLK family from *S. officinarum* was named *SoLRR-RLK1* ~ *SoLRR-RLK312* according to the physical location on the chromosome. Based on their chromosomal locations, 288 *SsLRR-RLK* genes were also named *SsLRR-RLK1* ~ *SsLRR-RLK 288* in *S. spontaneum*. Finally, the physical and chemical properties of the *LRR-RLK* genes from two *Saccharum* species were calculated using TBtools software, including amino acid length (NA), molecular weight (NW), isoelectric point (PI), protein instability index (II), aliphatic index (AI), and grand average of hydropathicity (GRAVY).

### Phylogenetic analysis and classification of *LRR-RLK* gene family

Using the 312 *SoLRR-RLK* genes and 288 candidate *SsLRR-RLK* genes identified as described above, a phylogenetic tree was constructed using Fastree software. And two *Saccharum* species LRR-RLK family was classified into 15 subfamilies (LRR I-LRR XV) based on the classification of *Arabidopsis thaliana* LRR-RLK family members. The plant phylogeny tree including *Fragaria vesca*, *Malus domestica*, *Pyrus bretschneideri*, *and Prunus persica* [41], *citrus clementina and Citrus sinensis* [76], *Solanum lycopersicum* [14], *Solanum tuberosum* [14], *Arabidopsis thaliana* [77], *Glycine max* [78], *Medicago truncatula* [15], *Oryza sativa* [12], *Physcomitrium patens* [4], etc. all 18 plant species with *S. spontaneum* and *S. officinarum* were constructed by the TimeTree database website (http://www.timetree.org)*.*

### Cis-acting regulatory elements analysis of *Saccharum LRR-RLK* genes

The upstream 2000 bp promoter sequences of the *SoLRR-RLK* gene were extracted for further analysis. The cis-acting elements in the promoter were predicted using the database PlantCARE (http://bioinformatics.psb.ugent.be/webtools/plantcare/htmL/). Only the typical and functional cis-acting elements were retained, and those that were ubiquitous in most genes, such as CAAT-box, TATA-box, TATC-box, and others, were filtered out.

### Synteny, Gene duplication and selection pressure analysis of LRR-RLK family

According to the genome annotation information of two *Saccharum* species, the chromosomal position information of the *LRR-RLK* genes were obtained. And the Synteny analysis was performed using BLASTP software and MCScanX software. To visualize the results, we employed circos v 0.69–8 software to map the *LRR-RLK* genes onto the corresponding chromosomes and depict the intraspecific gene collinearity. The collinearity between sugarcane (*S. spontaneum* and *S. officinarum*) and sorghum was analyzed and visualized using JCVI software. The Ka/Ks ratio between homologous genes of these species was calculated using Ka/Ks_Calculator Version:2.0 software to assess the degree of selective pressure and evolutionary divergence. And the divergence time (T) was calculated using the formula T = Ks/(2 × 6.1 × 10^–9^) × 10^–6^ [79].

### Analysis of expression patterns of *Saccharum* LRR-RLK family

The cDNA libraries were prepared according to the manufacturer's protocol (TruSeq® RNA, Illumina). RNA-seq libraries were pooled and sequenced at 100 nt paired-end on an Illumina HiSeq2500 platform at the Centre for Genomics and Biotechnology, Fujian Agriculture and Forestry University. Raw data were aligned to reference gene models (sorghum gene models) using TRINITY. Furthermore, Quantitative analysis of relevant RNA-seq data was conducted using Trinity Transcript Quantification, a method that involves aligning sequenced reads to annotated CDS sequences and employing the RSEM algorithm to compute the Fragments Per Kilobase of transcript per Million mapped reads (FPKM) values for each gene. The detailed methodology can be found at the official GitHub repository (https://github.com/trinityrnaseq/trinityrnaseq/wiki/trinity-Transscript-Quantification). Using pheatmap and TCseq R packages to draw heatmaps and trend cluster analysis, respectively.

### Supplementary Information

**Additional file 1.** Members of identified *LRR-RLK* gene family in *S. spontaneum* and *S. officinarum.*

**Additional file 2.** Members of identified *LRR-RLK *gene family in Sorghum and Arabidopsis.

**Additional file 3.** The physical and chemical information of *S. spontaneum* and *S. officinarum.* *LRR-RLK* genes.

**Additional file 4.** The classification of the LRR-RLK family by phylogenetic tree.

**Additional file 5.** Collinear gene pairs in the *LRR-RLK* gene family.

**Additional file 6.** The replication type of *LRR-RLK* gene in two founding* Saccharum*.

**Additional file 7.** Divergence time estimation for the orthologous genes of LRR-RLK from *Sorghum bicolor* and *Saccharum.*

**Additional file 8.** The expression value of *SoLRR-RLK* genes in both temporal and spatial models. FPKM values of *LRR-RLK* genes across tissue and developmental stages, leaf developmental gradient, and circadian rhythm in *S. officinarum*. Sd: Seedling stage; PM: Pre-mature; M: Mature; SL: Seeding leaf; SS: Seeding stem; LR: Leaf roll; LF: Leaf; BZ: Basal zone; TZ: Translational zone; MZ1: Maturing zone 1; MZ2: Maturing zone 2.

**Additional file 9.** The expression value of *SsLRR-RLK* genes in both temporal and spatial models. FPKM values of *LRR-RLK* genes across tissue and developmental stages, leaf developmental gradient, and circadian rhythm in *S. spontaneum*. Sd: Seedling stage; PM: Pre-mature; M: Mature; SL: Seeding leaf; SS: Seeding stem; LR: Leaf roll; LF: Leaf; BZ: Basal zone; TZ: Translational zone; MZ1: Maturing zone 1; MZ2: Maturing zone 2.

**Additional file 10.** Expression of *LRR-RLK* genes in *Saccharum* infected with different disease. P1: CK; P2: inchoate; P3: advanced. CK1: +1 leaf control; CK2: -3 leaf control; S1: +1 leaf infection; S2: -3 leaf infection.

**Additional file 11.** The expression trends of LRR-RLK family in tissue and developmental gradient. Sd: Seedling stage; PM: Pre-mature; M: Mature; SL: Seeding leaf; SS: Seeding stem; LR: Leaf roll; LF: Leaf; BZ: Basal zone; TZ: Translational zone; MZ1: Maturing zone 1; MZ2: Maturing zone 2.

**Additional file 12.** The expression trends of LRR-RLK family in *Saccharum* infected with different disease.

**Additional file 13.** The primers used for RT-qPCR.

## Data Availability

All data generated or analyzed in the course of this study have been comprehensively documented in the supplementary information files. The genomic data employed for testing purposes in both sugarcane were sourced from the autopolyploid Saccharum Genome (http://www.zhangjisenlab.cn/resource/genomic/28715.html) and the *S.bicolor* genome (https://phytozome-next.jgi.doe.gov/info/Sbicolor_v3_1_1), respectively. To determine the domain architecture of the *LRR-RLK* genes, we referred to the Pfam database (http://pfam.xfam.org/). Specifically, for investigating sugarcane pokkah boeng disease, we extracted relevant sequencing data from SRP127969 (https://www.ncbi.nlm.nih.gov/sra/SRP127969), while data pertinent to sugarcane mosaic virus disease were drawn from SRR10058145, SRR10058144, and SUB12324308 available in the GenBank database. Additionally, RNA-seq data encompassing different tissues of developmental stages, leaf segments, and circadian rhythms were sourced from the sugarcane public database (http://sugarcane.zhangjisenlab.cn/sgd/html/mRNA.html).
