# Supplementary figures and images for: Genomic investigation of duplication, functional conservation, and divergence in the LRR-RLK Family of Saccharum
Source: BMC Genomics. 2024 Feb 9;25:165. doi: 10.1186/s12864-024-10073-z (PMC10854099; doi:10.1186/s12864-024-10073-z)

a

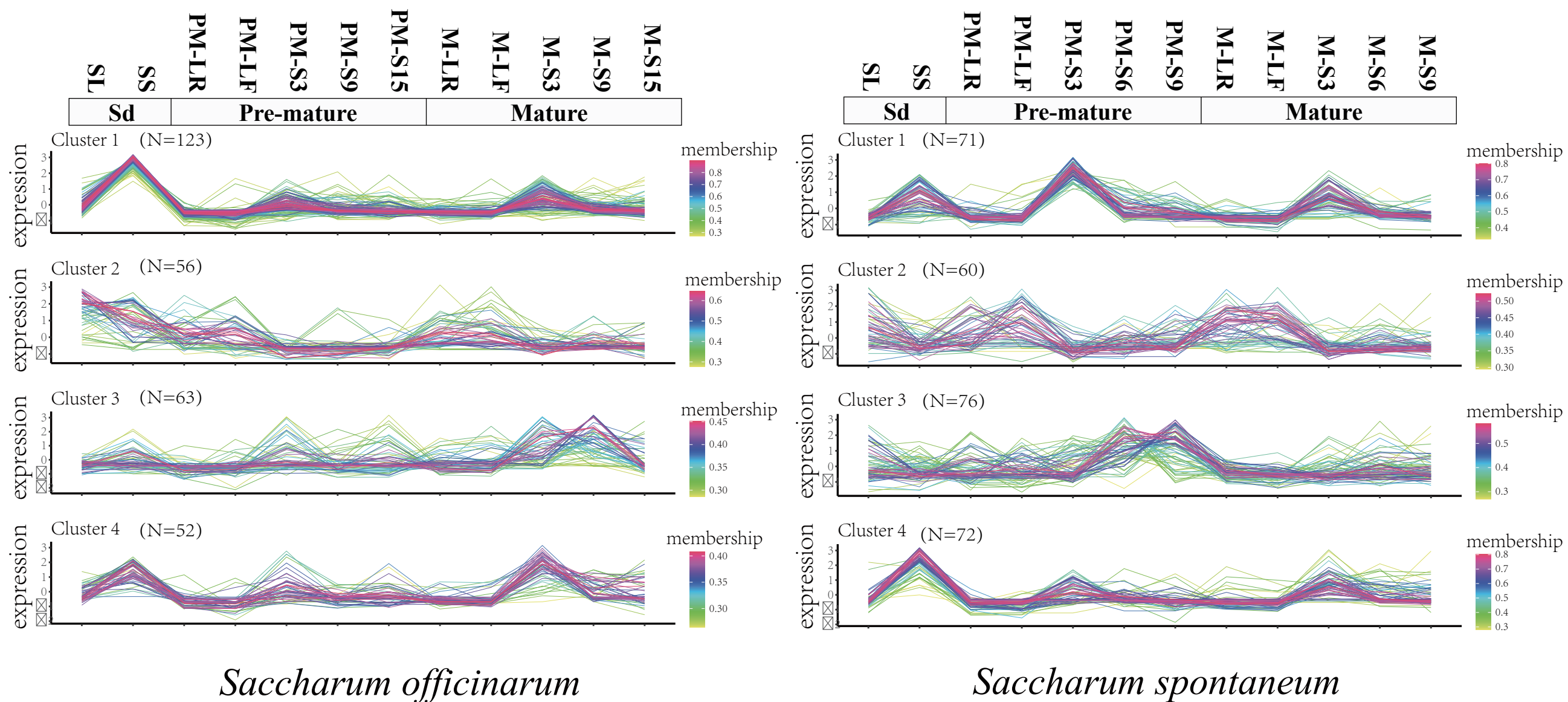

b

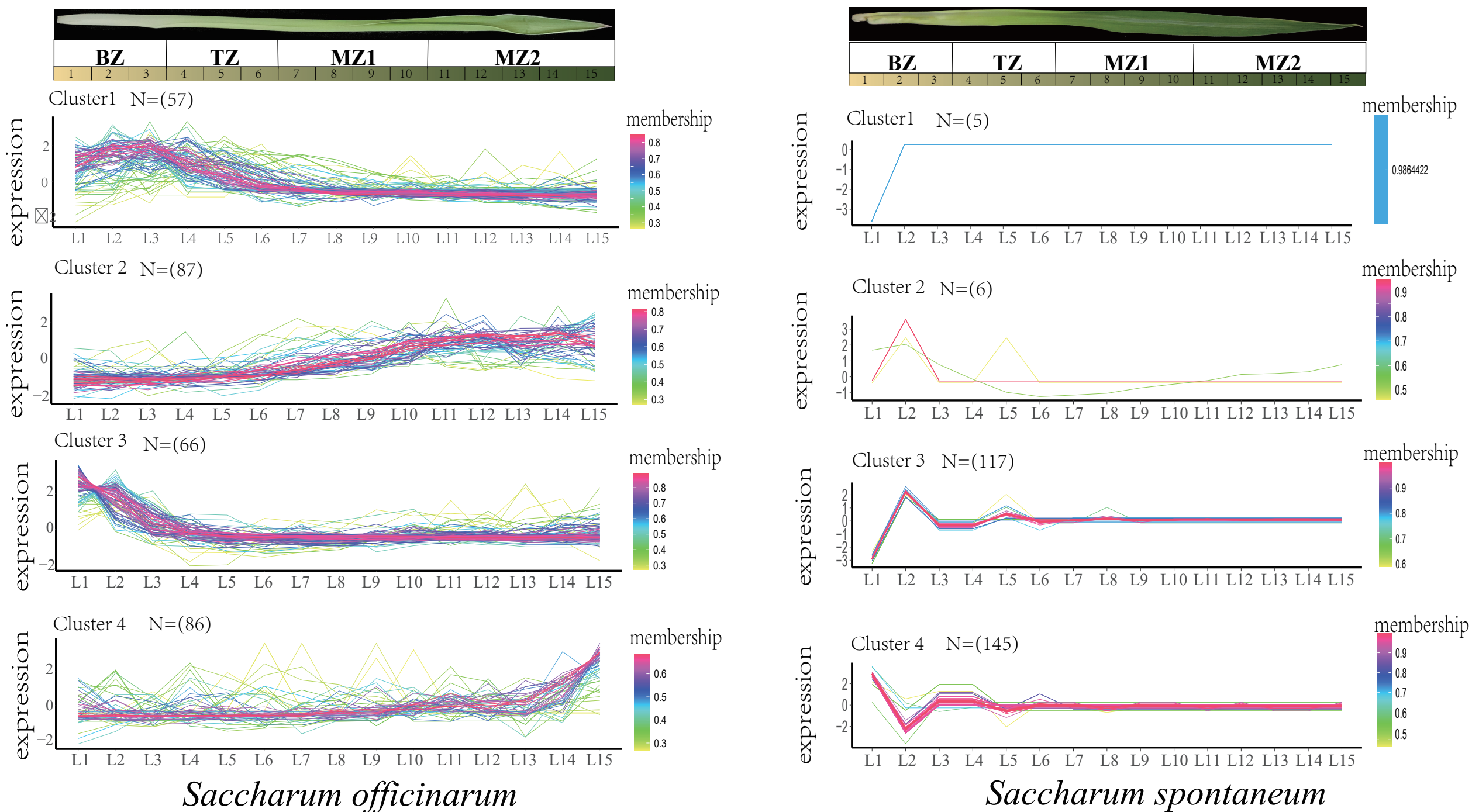

Supplement: Supplementary file 11 — Additional file 11. The expression trends of LRR-RLK family in tissue and developmental gradient. Sd: Seedling stage; PM: Pre-mature; M: Mature; SL: Seeding leaf; SS: Seeding stem; LR: Leaf roll; LF: Leaf; BZ: Basal zone; TZ: Translational zone; MZ1: Maturing zone 1; MZ2: Maturing zone 2. [file 12864_2024_10073_MOESM11_ESM.pdf]

# Sugarcane mosaic disease

**a**

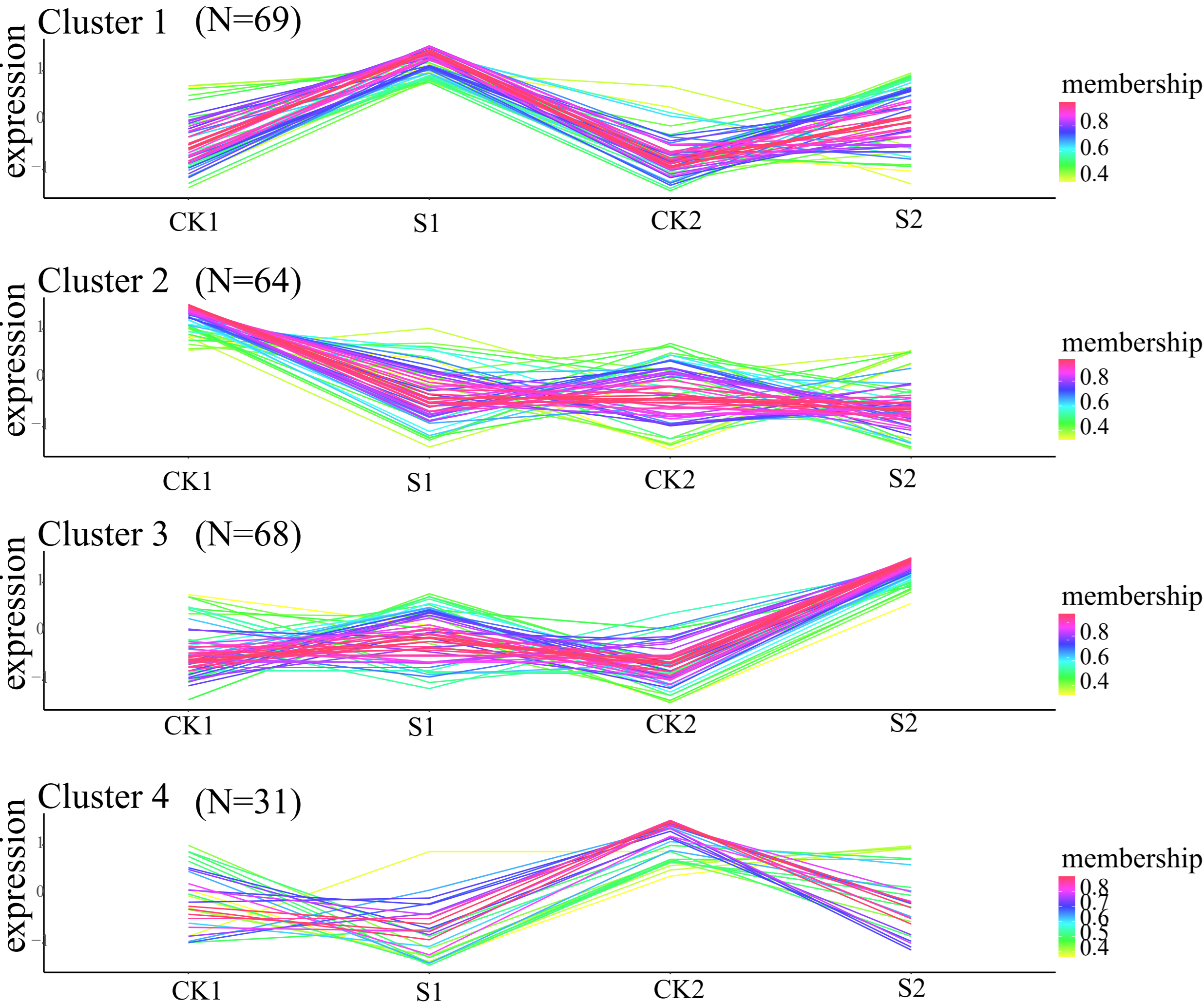

# Pokkah boeng disease

**b**

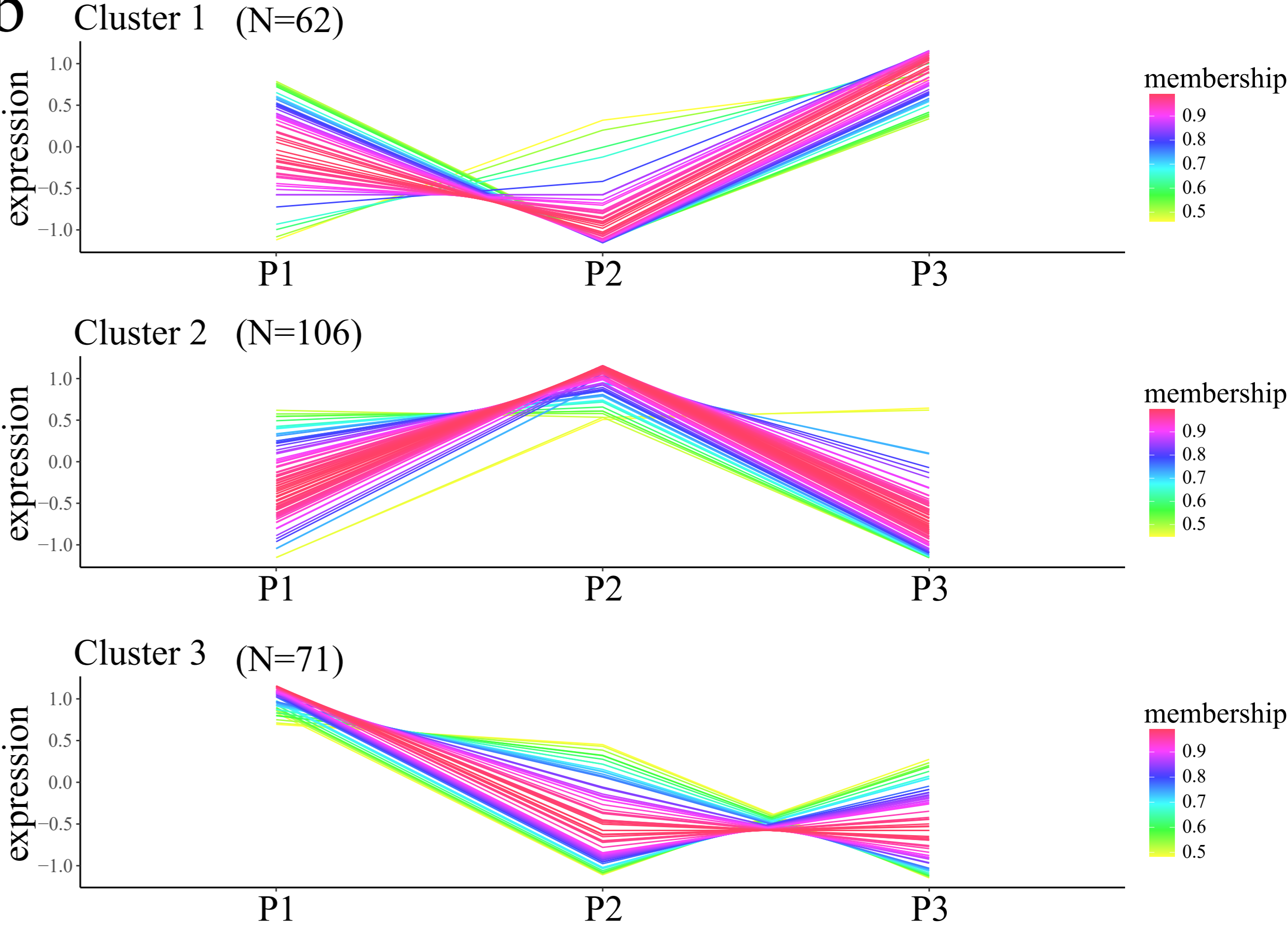

Supplement: Supplementary file 12 — Additional file 12. The expression trends of LRR-RLK family in Saccharum infected with different disease. [file 12864_2024_10073_MOESM12_ESM.pdf]
